# Do fungi have an innate immune response? An NLR-based comparison to plant and animal immune systems

**DOI:** 10.1371/journal.ppat.1006578

**Published:** 2017-10-26

**Authors:** Jessie Uehling, Aurélie Deveau, Mathieu Paoletti

**Affiliations:** 1 Biology Department, Duke University, Durham, North Carolina, United States of America; 2 INRA Université de Lorraine, Interactions Arbres-Microorganismes, UMR 1136, Champenoux, France; 3 Institut de Biologie et Génétique Cellulaire, UMR 5095 CNRS et Université de Bordeaux, Bordeaux, France; The Sainsbury Laboratory, UNITED KINGDOM

## Introduction

Survival and evolutionary success of living organisms directly depend on the ability to respond appropriately to their biotic environments. Plants and animals have developed intricate mechanisms of recognition and response that require differentiation between self and potentially pathogenic nonself and containment of infected tissues [1]. A class of cytosolic Nucleotide Oligomerization Domain (NOD)-like receptors, or NLRs, contribute to this recognition and discrimination process in plants and animals [2,3]. Less is known about how fungi monitor their interactions with their biotic environments. Here, we summarize evidence indicating that fungi have similar NLR proteins and may use similar mechanisms to recognize and respond to heterospecific nonself; we outline similarities and differences with their plant and animal counterparts, and we propose future directions elucidating aspects of fungal immune systems.

## 1. Why should fungi identify nonself?

**Fig 1:**
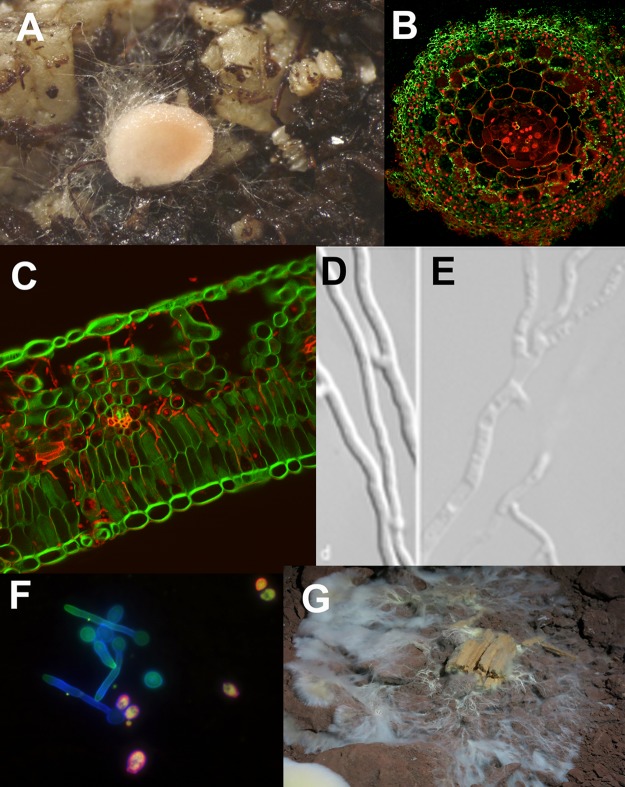
Diversity of fungal habitats and lifestyle leading to a large range of biotic interactions. a. Hyphae of the symbiotic fungus *Laccaria bicolor* and a fruiting body primordium surrounded by soil particles (INRA JL Churin); b. Transversal section of an ectomycorrhiza of the black truffle *Tuber melanosporum* (green) associated in symbiosis with a root of the tree *Carpinus betula* (red); c. The rust fungus *Melampsora larici populina* (red) invading a poplar leaf (green) (INRA S Hacquard, S Duplessis); d. Saprophyte fungus *Podospora anserina* growing freely; e. Saprophyte fungus *P*. *anserina* experiencing cell death during the interaction with bacteria of the genus *Serratia* (right panel); f. Dimorphic stages (i.e., yeast versus hyphae) of the human opportunistic pathogen *Candida albicans*; g. A wood-degrading fungus emerging from decaying wood and colonizing surrounding soil.

Fungi colonize nearly all environments on Earth (reviewed in [4]), where they interact with every major organismal group: viruses, bacteria, protists, amoeba, plants, and animals. Many of these interactions lead to symbioses ranging from mutualism to pathogenesis (Fig 1) [4]. Examples include fungi that are targeted by pathogens and predators [5] or that become hosts for intracellular bacterial populations [6]. Some of these fungal endosymbiont interactions have coevolved over their 400 million year old [7] symbiosis and likely involve specific bidirectional recognition systems [8–10]. The evolutionary success of the kingdom Fungi and diversity of fungal biotic interactions suggest that, like plants and animals, fungi have developed the ability to accurately identify and respond to interacting organisms. However, mechanisms for such monitoring and response are just beginning to be understood [11]. An intriguing possibility is the involvement of NLR-based nonself recognition mechanisms in Fungi.

Fungi possess mechanisms that allow for self and nonself recognition within species during vegetative development, including vegetative (heterokaryotic) incompatibility controlled by *het* (or *vic*) genes [12,13]. Vegetative incompatibility (VI) between genetically distinct mycelia helps maintain the integrity of mycelial individuals, permitting them to persist and evolve. VI also prevents the spread of mycoviruses (reviewed in [14]). In ascomycetes, some *het* genes involved in VI encode NLR proteins that contribute to allorecognition, but their involvement in xenorecognition is still largely unknown [15].

## 2. How do plant & animal NLR-based innate immune systems function?

**Fig 2:**
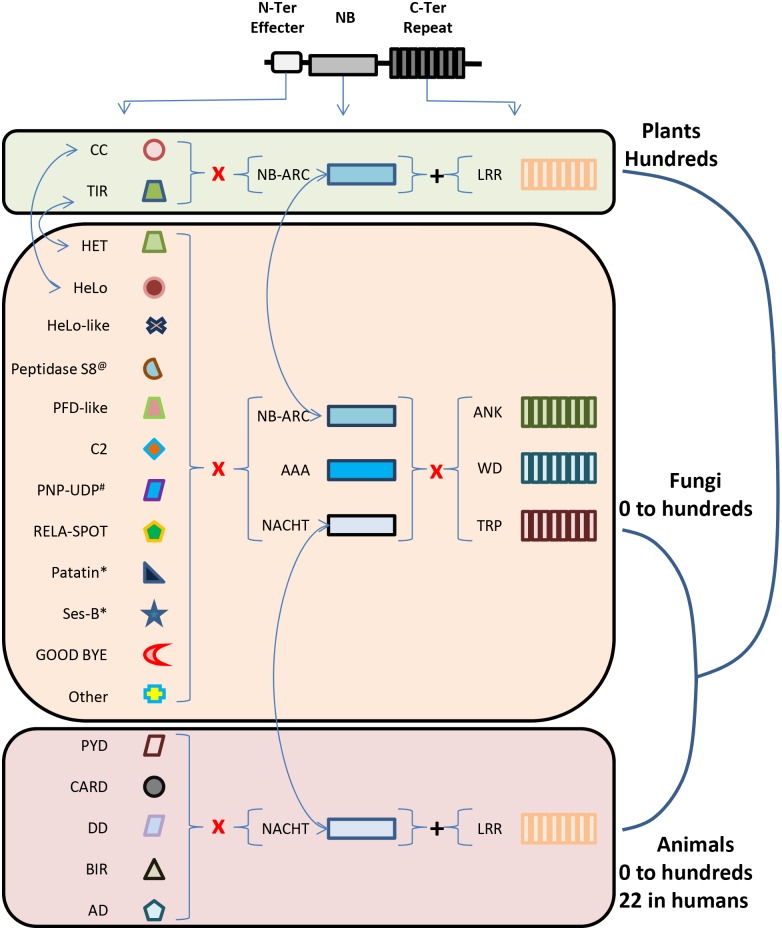
NLR domain diversity and abundance in plants, animals, and fungi. Only Pfam-A annotated domains are presented. Fungal NLR include NB domains found in plants and in metazoans. Three main types of repeated domains are found, but LRRs typically found in plant and animal NLRs are missing. All fungal effector domains are also found in non-NLR proteins. Some fungal effector domains have predicted enzymatic activities (* for lipases, ^@^ for proteases, ^#^ for UDP-phosphorylase). Almost all combinations of N-terminal effector domains, central NB domains, and C-terminal repeat domains can be found in fungal genomes. Arrows indicate domains related amongst branches of the eukaryotic kingdom. Abbreviations: LRR, leucine-rich repeat; NLR, nucleotide oligomerization domain-like receptors.

Innate immunity is an ancestral defense mode that involves microbial recognition, signal transmission, transcriptional reprogramming, and rapid cell death in animals, plants, and other organisms [16,17]. Plant and animal innate immune receptors, including NLRs, recognize pathogenic markers and initiate cell death. In plants, cell death occurs via the hypersensitive response, and in animals, cell death (pyrotopsis) occurs via inflammasome assembly [18]. Although the downstream signaling events and cellular death activation mechanisms differ between plants and animals, in both cases NLR activation results in rapid cell death and expression of antimicrobial compounds to restrict the spread of the pathogens. Similarly, VI in fungi leads to rapid cell death and increase of antimicrobial activity [19,20]. Plant and animal NLRs share similarities in structure and in pathogen-detection functions, although microbial molecules recognized by each group differ. Typical NLRs in both groups are tripartite, containing a central nucleotide-binding domain (NBD), an N-terminal domain that initiates downstream events, and a C-terminal Leucine-Rich Repeat (LRR) domain that facilitates protein–protein interactions [3,16]. The central NBD domains are related but can be differentiated, with NB-ARC and NACHT domains (named after proteins containing them) found in plant and animal NLRs, respectively (Fig 2). Although the N-terminal domains differ, C-terminal LRR domains are common to both groups. Fungal NLRs share similar structure and domain homology, further discussed below.

While some plant and animal NLRs detect pathogens directly, others monitor modified or damaged self, leading to indirect detection [16]. These observations led to the “Guard Model” for plants, in which a response is elicited when a pathogen effector disrupts a complex between the guardee (a host protein) and the guardian (an NLR) [21,22]. In the related “Decoy Model,” a duplicated guardee protein gains a novel function of luring effectors off target. Decoys may be integrated as an additional domain to the conventional tripartite organization of NLRs [23,24]. Although NLR structure, motif, and mechanistic functioning of NLR proteins are strikingly similar (Fig 2) [2], the evolution of NLRs in plants and animals is thought to be convergent [1,2,25]. How fungal NLRs are related to similar proteins in plants and animals and innate immunity is currently being investigated.

## 3. How do fungal NLRs mediate nonself recognition?

A subset of fungal VI genes with functionally validated roles in nonself recognition encodes NLR proteins [26–28]. The VI process dictates whether cells generated by the vegetative fusion of different conspecific fungal strains will develop or die after fusion [14]. Many fungal proteins involved in VI contain the cell death–inducing HET domains [28], and several are NLRs [26,29]. Fungal NLR activation during VI requires ligand-dependent activation [25], hydrolysis of nucleotides [30], and indirect evidence suggests oligomerization of NLRs [26,29]. In some cases, NLR signal transduction depends on amyloid fold transmission, requiring interactions with downstream effector proteins that are also observed in cell death activation mechanisms in metazoans [26]. Furthermore, a fungal NLR-associated amyloid-forming domain can functionally replace metazoan NLR effector domains, forming higher-order structures to ensure signal transduction [31]. During VI, incompatible fungal strains undergo programmed cell death, resulting from interactions between HET domain-containing proteins, NLRs, and others [13,26]. VI, including NLR-controlled VI, is thought to restrict horizontal spread of deleterious cytoplasmic elements or confine invasive pathogens, such as viruses [32]. Fungal NLRs controlling VI may also be involved in bacterial–fungal interactions [33,34] and potentially enable fungal detection of conserved molecular-associated patterns or microbe-associated molecular patterns (lipo-polysacharides, flagellin, peptidoglycan, etc.) or live bacteria [20]. It has also been hypothesized that VI evolved from a larger response to heterospecific nonself, leading to the idea that NLR-controlled VI emerged from a larger fungal innate immune system [12] similar to allorecognition responses in animals or hybrid necrosis in plants. Transcriptomic analyses of NLR-controlled VI [19,20,27] have revealed overlap with responses induced by bacteria and activation of processes comparable to plant or animal innate immunity, including autophagy [27], secondary metabolite production [11,27], and cell wall modification [11].

## 4. How diverse are fungal NLRs?

Most currently available fungal genomes encode a diverse repertoire of up to 200 (on average 30) NLRs [35]. Some fungal genomes (largely Saccharomycotina) do not encode NLRs, suggesting that fungal immunity involves additional mechanisms, a pattern shared by *Drosophila* and worm species [16]. Fungal NLR repertoires are highly variable and display intraspecific variation [35]. Fungal NLR structure is strikingly similar to plants and animals in being tripartite and containing C- and N-terminal domains flanking a central NB domain. In contrast to plant and animal NLRs that are assembled from relatively few domains, domain diversity in fungal NLRs includes at least 12 N-terminal effector domains, 3 central NB domains, and 3 C-terminal repeat Pfam domains (Fig 2) [35]. Fungal NLRs contain both the plant- and animal-like central NB domains (NB-ARC and NACHT, respectively), with observations of animal-like NACHT NB domains being more frequent (Fig 2). There are many open research avenues regarding fungal NLR functioning because only half of the N- or C-terminal domains have Pfam-A annotations [35]. Also, in contrast to plant and animal NLRs, some fungal N-terminal effector domains have predicted enzymatic activities (Fig 2) or pore-forming toxin properties [26], suggesting an autonomous mode of action rather than signaling function. Although not well documented, some fungal NLRs include unusual domains that might act as decoy domains, as highlighted in plant NLRs’ integrated decoy. For instance, a *P*. *anserina* NLR protein includes a zinc finger DNA-binding domain in its C-terminal region (personal observations).

Fungal NLRs include domains that share homology with plant or animal NLR effector domains. For instance, homology searches show similarity between the fungal HET domain and the TIR domain [35], as well as between the fungal HeLo-like domain and the N-terminal part of plant CC-type NLRs [29]. Similarities in eukaryotic NLR diversity, domain architecture, and functioning suggest that fungal NLRs are analogous to plant and animal innate immune receptors, combining features of both [35,36]. Plant and animal NLRs are thought to have arisen by convergent evolution [1,36], and the central NB domains of fungal NLRs analyzed so far support the view that NLRs are similar because of convergence [1]. It is tempting to hypothesize that fungal NLRs have similarly converged on tripartite structure to facilitate an immune response, particularly given the experimental data in plant and animal systems [16], demonstrating the ease and elegance of NLR-based molecular switches for immunity.

## 5. What role do fungal NLRs play in fungal innate immunity?

We are just beginning to understand the breadth of fungal NLR diversity and functioning. Elucidating the role of NLRs and other fungal immunity genes will mean addressing the following questions:

- **What signals are recognized by fungal NLRs?**
  Identifying signals that candidate NLRs respond to could be accomplished by developing protein interaction assays to define complex constituents in the presence and absence of pathogens.
- **How are NLR responses initiated?**
  Similarities between N-terminal effector and central NBD domains suggest that fungal NLRs function in multimeric complexes, such as in animals and plants [3,37], and recruit downstream activators of the pathways. In vitro and heterologous protein pull-downs may identify NLR superstructure binding partners. Interestingly, some fungal NLR effector domains have predicted enzymatic activities (Fig 2). These NLRs might function alone to initiate a response. Confirming these activities and identifying their substrates will be of interest.
- **What genes are controlled by fungal NLRs?**
  Comparative genomics and transcriptomics would offer insight into fungal immunity by identifying genes and proteins that respond to NLR-based immune activation. Further gene expression analyses in NLR loss- or gain-of-function mutants may be useful to decipher specific NLR responses. Once identified, producing fluorescent fusions with NLR-activated genes and monitoring protein expression and localization could also provide indications on the nature of the induced response.
- **How do NLR genes evolve?**
  Employing population genomics and selection analyses will help identify components of fungal immunity, including candidate NLR genes under varying selective regimes [28], such as the conservation of classic MAMP-detecting NLRs.

## 6. Fungi as promising models to study NLR-based response to nonself in eukaryotes?

NLR functioning in plants and animals provides pathogen detection and interspecific communication [2], such as maintenance of the host microbiome equilibrium [38]. Many fungi also host bacterial communities, which increase fitness and functioning of fungal metaorganisms. In this sense, fungal systems provide an exciting opportunity to investigate many aspects of NLR-based responses, such as host–microbiome and host–pathogen interactions. Relative to plant and animal systems, fungi offer the following benefits: many currently studied fungi are easy to cultivate, they produce numerous individuals and generations that can be analyzed simultaneously, there are genetic manipulation tools available for many fungi, available population genomic data are increasing rapidly, and they are amenable to in vitro evolutionary studies.

Fungi are widely used in industry, food production, and medicine [4]. Elucidating NLR-based fungal communication mechanisms could simplify the production and purification of bioactive molecules by getting rid of the bacterial species initiating such products [39]. Finally, fungi are responsible for devastating diseases of plant crops and animals, including humans. As each fungal species develops its own NLR repertoire, understanding NLR-mediated fungal immunity in pathogenic fungi reveals specific targets for drug development to activate fungal cell death.
